# Iatrogenic cerebral proteinopathies—The dawn of transmissible neurodegeneration? The example of cerebral amyloid angiopathy

**DOI:** 10.3389/fneur.2025.1693566

**Published:** 2025-11-05

**Authors:** Matija Zupan, Bruno Splavski, Senta Frol

**Affiliations:** ^1^Department of Vascular Neurology, University Medical Centre Ljubljana, Ljubljana, Slovenia; ^2^Faculty of Medicine, University of Ljubljana, Ljubljana, Slovenia; ^3^Department of Neurosurgery, Dubrovnik General Hospital, Dubrovnik, Croatia; ^4^Faculty of Applied Health Sciences, University of Zagreb, Zagreb, Croatia

**Keywords:** cerebral proteinopathies, medicolegal, transmissible, neurodegeneration, opinion

## Introduction

Intracranial accumulation and propagation of misfolded proteins is characteristic of many neurodegenerative diseases. These proteins could transmit from person to person as rare events after long-term incubation. Several reports confirmed possible inadvertent iatrogenic intracranial transmission of amyloid-beta (Aβ) pathology via medical and surgical procedures (1, 2).

A common molecular mechanism, described in prion diseases, is proposed to trigger the replication and spread of different misfolded proteins in the central nervous system (CNS), causing different neurodegenerative diseases associated with disease-specific proteins such as α-synuclein in Parkinson's disease, and amyloid-beta (Aβ) in Alzheimer's disease, and cerebral amyloid angiopathy (CAA) (3). While CAA is not exclusively caused by Aβ, other non-Aβ proteins may also contribute to its emergence, and possibly also to the iatrogenic CAA transmissibility (4).

CAA is an age-related disorder characterized by the progressive deposition of Aβ in cortical and leptomeningeal blood vessels' walls and brain parenchyma (5). Symptoms include cognitive impairment, transient focal neurological episodes, while neuroradiology entails cortical microbleeds, subarachnoid hemorrhage, cerebral superficial siderosis, and a lobar intracerebral hemorrhage (ICH) location (5). Positron emission tomography (PET) is increasingly utilized to detect diffuse cortical amyloid deposits (6), while cerebrospinal fluid (CSF) analysis demonstrates lowered Aβ levels (7). The definitive diagnosis requires histopathological examination through intraoperative biopsy or post-mortem autopsy (8).

Iatrogenic cerebral amyloid angiopathy (iCAA) is a rare but increasingly recognized form of CAA, often presenting earlier than age-related cases, typically between the third and fifth decade. Its prevalence is ~4% in patients >50 and up to 45% in those < 55 (9). Patients commonly show cognitive decline, seizures, and recurrent lobar ICH (10). iCAA is linked to prior exposure to cadaveric dural grafts (e.g., Lyodura^®^) (11), pituitary-derived growth hormone (12), prion-contaminated neurosurgical instruments (9, 13), and possibly red blood cell (RBC) transfusions (14, 15).

Iatrogenic CAA shares pathophysiological mechanisms with prion diseases (16). Spatial correlation between prior neurosurgical sites and CAA pathology has been in favor of localized iatrogenic seeding (8, 17). Recently, the plausibility of Alzheimer-type pathology being transmissible in a prion-like fashion has been emphasized (18). Typically, there is a long latency between exposure and symptoms, often 30–40 years. Medical procedures involving exposure to Aβ beyond childhood have been associated with possible iCAA in elderly patients (19). The diagnostic criteria distinguish probable from possible iCAA based on relative certainty and imaging features (which, in contrast to the Boston Criteria Version 2.0, allow for some, but not predominantly, deep hemorrhagic imaging features) (10). Patients have probable iCAA if PET, CSF, or biopsy findings provide evidence of Aβ accumulation in the CNS and genetic causes are excluded. Possible iCAA is considered when supporting diagnostics are not available or performed, but there is a history of relevant exposure (10). Hence, diagnosing requires a detailed medical history to identify potential exposure to sources of Aβ. In the absence of disease-modifying treatment, current management is aimed at preventing ICH recurrence with antihypertensive management and avoidance of anticoagulation treatment (5).

To date, more than 125 patients with presumed iCAA have been identified, mainly related to intracranial use of cadaveric dura (8, 9, 11, 20, 21).

Historically, the understanding of amyloid transmission was limited to prion diseases such as iatrogenic Creutzfeldt-Jakob disease (iCJD), which is linked to unintentional exposure to prions during various medical and neurosurgical procedures (22). As iCAA joins iCJD as a preventable, procedure-associated disease, a paradigm shift in clinical, ethical, and medico-legal domains is warranted. The growing literature on iCAA raises critical, medico-legal, and ethical questions—particularly regarding organ and tissue donor programs—and disclosure of diagnosis in clinical practice due to uncertainty about iCAA etiology, prognosis, and lack of treatments.

This opinion argues for an increased clinical, medico-legal, and ethical awareness of iCAA in younger patients presenting with atypical ICH.

## Discussion

Growing literature suggests that misfolded proteins, specifically Aβ, can be transmitted from person to person in a prion-like propagation through various medical and neurosurgical procedures. The appearance of Aβ seeding in the cerebral blood vessels a few decades after neurosurgical interventions indicates probable vascular complications associated with iCAA, including ICH, perivascular inflammation, and cognitive impairment (2).

### Disclosure of iCAA to the patient and their relatives

Disclosing the possibility of iCAA to a patient with symptoms compatible with CAA and a history of relevant exposure to exogenous Aβ conforms to the informed consent principle and is a prerequisite for respecting the patient's autonomy (23). In iCAA, it opens up relevant ethical considerations since this diagnosis is often associated with a certain uncertainty (20). Kaushik et al. (20) recently proposed three key ethical and clinical questions while dealing with iCAA patients: (i) whether clinicians should disclose suspicion of iCAA to patients, (ii) whether to recommend additional diagnostic testing to confirm or clarify the diagnosis, and (iii) whether to proactively identify and notify asymptomatic individuals at known risk due to prior exposure.

We advocate for cautious yet transparent communication—sharing diagnostic uncertainty while respecting patient autonomy and supporting informed decision-making—even in the absence of proven therapeutic benefit. Clinicians should discuss the possibility with patients who present with compatible clinical findings and have a relevant exposure history. This includes being upfront about uncertainties: while iCAA is a plausible consideration, it remains a rare diagnosis, and scientific understanding is still evolving. Because the prospect of an acquired, prion-like, incurable brain disease can be distressing (24), early integration of neurology, ethics, and mental health expertise into the disclosure process is essential.

Recommending further diagnostics seems justified, although treatment options remain limited. Diagnostic clarification can exclude alternative conditions, inform prognosis, and guide clinical decision-making, such as avoiding anticoagulation treatment and seeking alternative solutions and lifestyle counseling, emphasizing blood pressure control. Currently, the ICH recurrence rate in iCAA appears to be higher than in sporadic CAA, although this might reflect reporting bias (9). Hence, the benefit of early intervention might be greater in iatrogenic than sporadic CAA (25). There may also be a scientific benefit to disclosure because it might increase the willingness of patients to participate in scientific studies (20). Nevertheless, our approach should remain patient-centered, weighing potential benefits against financial cost and psychological burden.

Even more complex is the issue of proactive identification and notification of asymptomatic recipients of transmissible Aß in cadaveric human materials who are currently unaware of their risk. Namely, unlike in hereditary disease, no test is available to exclude or unequivocally confirm iCAA, and key information, such as confirmation of exposure status, is often lacking (20). Notifying asymptomatic individuals with confirmed prior exposure is ethically appropriate. However, broader proactive identification of at-risk people who may be unaware of their exposure is ethically valuable but logistically challenging. Timely diagnosis at pre-symptomatic or early symptomatic stages might have consequences for antithrombotic and blood pressure management. Moreover, when a patient is diagnosed with iCAA, it's the clinician's duty to inform any relatives at risk due to similar exposure routes and any individuals known to have been exposed to the same source or procedure. This may require coordination with public health authorities to enable batch notifications and establish trace-back investigation within hospitals and public health agencies.

### Medicolegal aspects of donor-recipient screening practices and sterilization protocols

#### The shift toward dural synthetic materials: an ethical and practical imperative

The documented link between cadaveric dural grafts and iCAA makes exclusive use of synthetic alternatives ethically and clinically necessary. While autologous grafts remain valuable for small defects, their limited supply and applicability mean synthetic, non-biological, absorbable substitutes offer the safest, most practical solution. They eliminate the risk of transmissible diseases while maintaining reliable biomechanical properties. Institutions should discontinue cadaveric graft use, review surgical material policies, and track individuals previously exposed to high-risk grafts. This proactive approach aligns with *primum non nocere* and supports early identification of iCAA in at-risk populations.

#### Medicolegal aspects of donor-recipient screening practices and sterilization protocols

Given the burden of transmissible neurodegeneration, the donor eligibility must be urgently reconsidered. Patients with lobar ICH (possible/probable CAA) are often considered for corneal or organ donation, yet detailed medical histories concerning childhood neurosurgical procedures and RBC transfusions are frequently overlooked. The potential for these individuals to harbor transmissible Aβ pathology, even in the absence of overt neurodegenerative symptoms, presents a previously underappreciated risk to recipients.

Past concerns about iCJD transmission via neurosurgical instruments prompted rigorous sterilization protocols, including sodium hydroxide (NaOH) and hypochlorous acid (HOCl) rinsing, alkaline detergents, or prolonged autoclaving, and the increased use of disposable instruments (26, 27). However, whether these protocols sufficiently inactivate misfolded Aβ aggregates remains unresolved. This is particularly concerning in the context of emerging evidence that standard decontamination procedures may be insufficient to fully denature amyloid seeds, thereby posing a risk of iCAA transmission (17, 18, 28). In low-to-moderate-income countries, the non-availability of disposable, single-use surgical instruments may pose a problem linked with possible iCAA transmission. It also poses moral questions about the universal right to equal quality health care, regardless of the economic wellbeing of its participants.

In donor programs, this issue is ethically complex. Donor registries rarely capture data on historic neurosurgical interventions or transfusions received decades earlier. Yet, the symptoms of spontaneous lobar ICHs on neuroimaging—particularly in patients under 55—should prompt a more thorough evaluation.

## Future perspectives

Iatrogenic neurodegeneration is no longer a theoretical construct, and transmissible neurodegeneration has become “a new normal” in the field of neuroscience. As iCAA joins iCJD in preventable procedure-associated diseases, we call for an urgent paradigm shift in clinical, ethical, and medico-legal domains.

This also raises a meaningful clinical question of whom we should screen for iCAA and how to deal with the issue of disclosure of such information to patients and their families. We suggest performing a thorough clinical evaluation of patients younger than 65, with otherwise atypical ICH and/or other CAA manifestations, especially in those without conventional risk factors such as arterial hypertension. We strongly advocate for mandatory biopsy in case of neurosurgical ICH evacuation and performing additional work-up consisting of PET imaging or CSF analysis, and genetic testing where applicable. A meticulous history should include data on past exposure to transmissible Aβ sources such as cadaveric graft material, pituitary-derived human growth hormone, prion-contaminated neurosurgical instruments, and transfusions of RBCs.

As many ICH patients are potential tissue and organ donors, one should ask whether these patients may represent a possible source of Aβ transmission to the recipients, with a much broader medico-legal implications, spanning clinical neuroscience practice, sterilization protocols and use of cadaveric-origin grafts in neurosurgery, transplantation and transfusion medicine at large. While no formal guidelines currently mandate this, the medical community must begin asking: should we actively exclude potential donors with lobar ICH and a medical history suggesting possible exposure to amyloid-contaminated materials or instruments? This may reduce the future burden of iatrogenic cognitive impairment and hemorrhagic complications in recipients. Furthermore, patients undergoing neurosurgical procedures with potentially contaminated transplants and instruments are obliged to receive long-term risk assessment, including systematic ethical considerations. The clinicians should emphasize the possibility of prion-like diseases to alleviate the risk of iatrogenic transmission and recognize the need for ongoing iCAA surveillance. We argue for enhanced international collaborations in the iCAA research to form a strong foundation for interdisciplinary guidelines on its management.

Multicentric long-term studies are needed to better understand potential routes of Aβ transmission and to clarify whether other similar intracranial proteinopathies, such as those caused by α-synuclein, can also be transferred iatrogenically (1).

Until more definitive risk-stratification tools are available, heightened awareness, careful donor/recipient selection, and transparent disclosure practices are essential to mitigate these risks.

## Conclusions

iCAA marks the emergence of transmissible neurodegeneration, calling for a paradigm shift in clinical, ethical, and medico-legal management. Collaborative guidelines across neurology, neurosurgery, infectious diseases, and transplant medicine are urgently needed to mitigate transmission risks, ensure transparency, and uphold moral responsibility. Medico-legal considerations regarding donor and recipient eligibility may require stricter regulations, broader awareness of iatrogenic transmissible diseases, and equitable access to healthcare for all patients and recipients, irrespective of social status or economic rank of their societies.
